# AI-enabled ChatGPT’s carbon footprint and its use in the healthcare sector: a coin has two sides

**DOI:** 10.1097/JS9.0000000000000905

**Published:** 2023-11-21

**Authors:** Chiranjib Chakraborty, Soumen Pal, Manojit Bhattacharya, Md. Aminul Islam

**Affiliations:** a Department of Biotechnology, School of Life Science and Biotechnology, Adamas University, Kolkata, West Bengal; b School of Mechanical Engineering, Vellore Institute of Technology, Vellore, Tamil Nadu; c Department of Zoology, Fakir Mohan University, Vyasa Vihar, Balasore, Odisha, India; d COVID-19 Diagnostic Lab, Department of Microbiology, Noakhali Science and Technology University, Noakhali; e Advanced Molecular Lab, Department of Microbiology, President Abdul Hamid Medical College, Karimganj, Kishoreganj, Bangladesh

*Dear Editor*,

A recent article by Wu and Zhang informed this journal that artificial intelligence (AI)-enabled ChatGPT helps advance healthcare and medicine^1^. Similarly, we highlighted ChatGPT’s role in the next-generation drug discovery and development in this journal. At the same time, we also suggested that pharmaceutical companies can apply this chatbot in therapeutics discovery and development^2^. Therefore, ChatGPT has a broader range of applications. Researchers are applying ChatGPT in various fields, from biology to medicine. Concurrently, it indicates that the AI-enabled ChatGPT application is growing speedily day by day. Therefore, these two articles published in this journal are significant and timely.

Presently, the increasing use of digital technologies (DT) and AI is increasing carbon emissions in the atmosphere. Several researchers have shown their concern in this direction. Recently, An *et al*. described that the use of generative AI and LLM (large language model) is increasing regularly. At the same time, different companies such as Google, Meta, and Microsoft are investing billions of dollars in the area of generative AI or LLM, like ChatGPT. It has been noted that although LLM can produce intelligent text, electricity consumption is higher. Researchers reported that the consumption of electricity in the case of the present LLM is much higher compared to previous versions^3^. The requirement of energy in several Kilowatt during the training for various NLP (natural language processing) models can be calculated presently using other models. Recently, Dhar reported that the carbon footprint for training a single LLM is too high for CO_2_ emissions, which might equal around 300000 kg of CO_2_ emissions. It has been described as 125 round-trip flights between two distant cities, namely New York and Beijing^4^. Similarly, it has been reported that cloud computing consumes ~0.5% of the world’s total energy consumption, which will grow in the coming years. It is projected that, in the coming years, this will grow beyond 2% of the world’s total energy^5^. However, it is time to understand how accurate the LLM carbon footprints are. To estimate the carbon footprint of machine learning (ML), a tool has been developed, which is called a ML emissions calculator. This tool considers several factors, such as the span of training time, the energy consumed by the particular system, the server’s geographical location (by cloud computing server), and the CO_2_ emissions per unit of electricity^5^. We need to develop a better model to compute the carbon footprint of AI products.

For the total greenhouse gas (GHG) emission or carbon footprint, the healthcare sector is one of the significant contributors. Patient transport is an essential component for moving from one place to another. As the same time it has been noted that carbon emissions can be a substantial factor in patient transport. Dacones *et al*. have computed transportation-related GHG emissions, especially for patient transport. They noted that outpatient visits augmented the carbon footprint emissions by 3.2% annually^6^. However, AI can minimize the excessive utilization of transport ultimately the carbon footprint, in the healthcare sector. This trend in the self-diagnosis was noted while using the internet. A survey by Kuehn informed that, among the U.S. population, more than one-third of people have gone through self-diagnosis using the internet^7,8^. Another study by Al Muammar *et al*. informed that the importance of internet use for different health information in the Middle East is rising regularly among the Saudi Arabian population^8,9^. Similarly, other researchers reported that several people have searched for information about various specific disease and its symptoms using the internet^10^. Therefore, it is evident that people are now regularly using the internet for self-diagnosis to get different information about health and disease symptoms. Interestingly, after ChatGPT’s introduction, this healthcare-related information fetching and self-diagnosis is increasing every day, and it helps reduce the number of remote patients going to the hospital. At the same time, telemedicine has provided an added advantage to the patients. It has been noted that after the introduction of telemedicine and AI, the number of remote patients in the hospital is decreasing^11^. This helps to reduce patient transport to the hospital incredibly, thereby reducing the carbon footprint. Recently, Wolf *et al*. stated that telemedicine significantly diminishes travel for patients and AI is likely to reduce the carbon footprint in the healthcare sector, and also estimated that about 80% decline in the carbon footprint might be achievable^12^. Researchers have tried to compute the increase in CO_2_ emissions during AI use by the patients, and the reduction in CO_2_ emissions due to a reduction in the patients’ travel. Finally, they compared the two cases and found a significant reduction in CO_2_ emissions. Similarly, the use of AI-enabled ChatGPT by remote patients will lower in-person referrals in the hospital. It might help significantly to reduce the transport as well as the carbon footprint. Hence, the use of ChatGPT by remote patients will be beneficial.

From the above discussion, it is apparent that AI-enabled ChatGPT might increase the carbon footprint in general cases. On the other hand, the LLM or ChatGPT in the healthcare sector could be beneficial in terms of carbon footprint, and it will help to reduce it. Therefore, ChatGPT has two different effects, although it deals with the same situation (carbon footprint). However, AI users should be urged to employ clean energy sources during its operation. At the same time, we also urge researchers to develop energy-friendly and energy-efficient hardware that might reduce the carbon footprint when utilizing AI.

## Ethical approval

None.

## Sources of funding

None.

## Author contribution

C.C.: conceptualization, data curation, investigation, and writing – original draft, review, and editing; S.P.: validation and editing; M.B. and M.A.I.: validation.

## Conflicts of interest disclosure

All authors report no conflicts of interest relevant to this article.

## Research registration unique identifying number (UIN)

1. Name of the registry: not applicable.
2. Unique identifying number or registration ID: not applicable.
3. Hyperlink to your specific registration (must be publicly accessible and will be checked): not applicable.

## Guarantor

Md. Aminul Islam, COVID-19 Diagnostic Lab, Department of Microbiology, Noakhali Science and Technology University, Noakhali 3814, Bangladesh; e-mail: aminulmbg@gmail.com.

## Data availability statement

The data in this correspondence article is not sensitive in nature and is accessible in the public domain. The data is therefore available and not of a confidential nature.

## Provenance and peer review

Not commissioned, internally peer-reviewed.
